# Editorial: Women in veterinary experimental and diagnostic pathology: 2021

**DOI:** 10.3389/fvets.2023.1190361

**Published:** 2023-04-12

**Authors:** Raquel Manzano, Inmaculada Martín-Burriel

**Affiliations:** ^1^Departamento de Anatomía, Embriología y Genética Animal, Facultad de Veterinaria, Universidad de Zaragoza, Instituto Agroalimentario de Aragón-IA2, Insitituto de Investigación Sanitaria de Aragón-IIS Aragón, Zaragoza, Spain; ^2^Centro de Investigación Biomédica en Red de Enfermedades Neurodegenerativas (CIBERNED), Zaragoza, Spain

**Keywords:** cerebrospinal fluid (CSF), biomarker, DNA methylation, prion, saliva, pig disorder, urothelial carcinoma, microRNA

The percentage of women participating in different professional sectors has risen over the last decades. The gender dimension is attracting special attention worldwide, and special measures are being undertaken to promote the incorporation and recognition of the contribution of women to different fields and levels, including legislation and policies. In line with this, initiatives such as the Gender Equality Strategy, sponsored by the EU, or the National Strategy on Gender Equity and Equality in USA reflect the raising awareness of governments and stakeholders. In this sense, Frontiers in Veterinary Science launched a series of Research Topics focused on gender perspectives in different areas of veterinary practice and research, which include the current *Women in veterinary experimental and diagnostic pathology: 2021*.

According to the last FVE (Federation of Veterinarians in Europe) survey, veterinary practice in Europe is becoming feminized, with an average of 58% of professionals being women, although this data varies greatly depending on the country, ranging from 35 to 80%. Despite these numbers, the pay gap is still relevant, with 12% less income being registered for veterinary women (http://fvesurvey.com/).

Likewise, academic careers in veterinary science are clearly biased in favor of either men or women depending on the level of seniority and responsibility, with similar tendencies occurring worldwide. It has been shown that while the representation of women at the associate lecturer level exceeded 75%, women corresponded to <30% of professorship appointments (1).

Similarly, according to UNESCO, <30% of researchers are women, and their participation in leadership research positions falls below 11%, with only 4% of Nobel Prizes having been awarded to women (2).

This Research Topic focuses on research performed in the field of veterinary pathology, from experimental to diagnostic approaches, whose main or senior authors are women.

Four original research manuscripts were approved for publication upon peer reviewing. To date, this Research Topic has received more than 8,700 viewings and 1,387 downloads.

The published manuscripts cover a range of veterinary pathology conditions and species, including equine, sheep, porcine and canine species.

The diagnosis of equine neurologic conditions represents a challenge due to the patient size, which limits the access to advanced imaging techniques and the collection of cerebrospinal fluids (CSF) for infection and cytology testing. Different sites for CSF collection have been reported, including the atlanto-occipital (AO), atlantoaxial (AA), and lumbosacral (LS) space. Centesis at the AA and LS sites is typically performed under standing sedation, while AO requires general anesthesia, with an exacerbated risk on recovery, especially for ataxic patients. There is thus a need for analysis of the CSF obtained from different centesis sites and their predictive potential for neurologic conditions and survival in equines. The study by Young et al.  includes a comparative assessment on the cytological characteristics of CSF collected from different puncture sites in >100 horses referred as suspects of neurological condition. The authors reported the robust correlation of cytologic abnormalities (particularly for total nucleated cell count) with poor survival and different cytologic read outs depending on the sample obtention site in unremarkable samples.

Scrapie, or prion disease, is an infective and fatal transmissible spongiform encephalopathy (TSE) affecting goats and sheep. Histologically, it is precisely defined by the accumulation of the pathogenic form of a glycoprotein called prion (PrP) in the CNS; however, neuropathologic cellular and molecular events need to be discerned. The study by Hernaiz et al. investigates epigenetic DNA methylation in the thalamus of sheep naturally infected with scrapie at a clinical stage using whole-genome bisulfite sequencing (WGBS). A total of 8,907 significant differentially methylated regions (DMRs) and 39 promoters (DMPs) were identified. The hypomethylated regions were enriched in transmembrane transport and cell adhesion genes, and hypermethylated regions were enriched in intracellular signal transduction genes. A quantitative PCR analysis of five of these genes containing DMR corroborated differential expression as compared to uninfected controls, matching the genomic study. This work suggests a potential regulatory role of DNA methylation in prion neuropathology.

Many of the commonly diagnosed pig disorders occurring in farms display concomitant stress and immune response activation, with saliva being the preferred sample to detect these parameters, as it is an efficient and low-invasive source. In this study, Sánchez et al. analyse a panel of 11 saliva biochemical parameters associated with psychosocial stress, adaptative immunity, and oxidative stress as predictive biomarkers to discriminate between different multifactorial disorders in pigs, namely, rectal prolapse, tail-biting lesions, diarrhea, lameness, and dyspnoea. These authors describe high associations between oxidative stress markers and adaptive immune markers and ≥4 parameters differentially regulated in each disorder analyzed. Lameness and rectal prolapse were the conditions affecting saliva parameters the most, while tail-biting lesions- and diarrhea-suffering specimens showed saliva patterns closer to that of healthy individuals. A principal component analysis managed to explain the 48.3% of data variance, which suggests the potential of biomarker saliva testing for pig disease diagnosis and discrimination.

Urothelial carcinoma (UC) is the most prevalent neoplasia affecting urinary tract, with a 50% lethality rate. Diagnosis of UC in dogs and humans is currently performed by histologic examination of the affected tissues, which encompasses the performing of a surgical biopsy. There is, therefore, an urgent need for low-invasive and specific diagnostic tools in the treatment of this disorder. MicroRNAs (miRNAs) are short non-coding RNAs, 21–25 nt long, that are expressed by most tissues and differentially regulated in distinct disorders, including neoplasia. In this study, Varvil et al. evaluate the miRNA profile in bladder tissue from canine normal urothelium and invasive UC using next-generation RNA sequencing (RNA-Seq). Differential expression was observed in 28 miRNAs, and the RT-qPCR confirmed that four miRNAs were significantly downregulated in the UC samples. Differentially expressed miRNAs were frequently associated with gene silencing, cancer, and DNA damage responses. The observed distinct miRNA pattern between the normal and UC canine bladder proves their potential role as diagnostic and therapeutic targets in this disorder.

The editors feel confident that this Research Topic will serve to highlight the impact and raise awareness of the work being carried out by women researchers in the veterinary field, strengthen their collaborative network, and represent a benchmark for younger veterinary women and researchers. A grateful recognition is displayed to all the authors and reviewers whose contributions were invaluable to the launch of “*Women in veterinary experimental and diagnostic pathology: 2021*.”

## Author contributions

All authors listed have made a substantial, direct, and intellectual contribution to the work and approved it for publication.

## References

[1] Liu X Dunlop R Allavena R Palmieri C. Women representation and gender equality in different academic levels in veterinary science. Vet Sci. (2021) 8:159. 10.3390/vetsci8080159 34437481 PMC8402655

[2] UNESCO UIS. Data for the Sustainable Development Goals. (2023). Available online at: http://uis.unesco.org/ (accessed April 5, 2023).

